# NLCECA score: a serum inflammatory-tumor biomarker score to predict survival of advanced perihilar cholangiocarcinoma after hepatic arterial infusion chemotherapy

**DOI:** 10.1038/s41598-024-53883-7

**Published:** 2024-02-23

**Authors:** Shjie Fu, Jie Li, Hua Fan, Kanglian Zheng, Boyu Leng, Guang Cao, Liang Xu, Yujie Zhong, Chuanxin Niu, Xiaodong Wang

**Affiliations:** 1 Key Laboratory of Carcinogenesis and Translational Research (Ministry of Education/Beijing), Department of Interventional Oncology, Peking University Cancer Hospital & Institute, Beijing, 100142 China; 2 Center for Medical Device Evaluation, National Medical Products Administration, Beijing, 100053 China; 3 Key Laboratory of Carcinogenesis and Translational Research (Ministry of Education/Beijing), Department of Cancer Epidemiology, Peking University Cancer Hospital & Institute, Beijing, 100142 China; 4 Hebei North University, Hebei, 075000 China

**Keywords:** Cancer therapy, Tumour biomarkers

## Abstract

Prognostic features in advanced perihilar cholangiocarcinoma (pCCA) patients who received first-line hepatic arterial infusion chemotherapy (HAIC) are unknown. The purpose of our study was to develop an applicable score based on serum inflammatory-tumor biomarkers to predict the survival of advanced pCCA patients who received first-line HAIC. In total, 106 advanced pCCA patients were enrolled as the training cohort. The optimal cutoff values of baseline variables were defined by the receiver operating characteristic method or according to previous publications. According to the results of Cox regression analysis, baseline neutrophil-to-lymphocyte ratio (NLR) > 3.19, carcinoembryonic antigen (CEA) > 10 ng/mL, and carbohydrate antigen 19-9 (CA19-9) > 200 U/mL were identified as independent survival predictors, which were used to develop the NLCECA score (NLR, CEA, and CA19-9). When including the NLCECA score in the multivariate analysis, the NLCECA score was the only independent predictor of survival. The risk of survival decreased by 111.9% for each 1-point increase in the NLCECA score. Additionally, the NLCECA score could also predict survival in another 33 patients in the validation cohort (*P* < 0.001). In summary, the NLCECA score is a potential biomarker system for predicting the survival of advanced pCCA patients who received first-line HAIC.

## Introduction

Cholangiocarcinoma (CCA) consists of intrahepatic (iCCA), perihilar (pCCA), and distal (dCCA) CCA^1^. pCCA, localized to the perihilar bile duct, is the most common, accounting for approximately 50% of all CCA cases^2^. However, when diagnosed, most pCCA patients are at the advanced stage, and thus without the opportunity for resection, resulting in dismal survival expectations^1^. Currently, the first-line treatment for advanced pCCA is systemic chemotherapy using gemcitabine and cisplatin (CisGem), with a median overall survival (mOS) of less than a year^3^. Hepatic arterial infusion chemotherapy (HAIC), which can increase local drug concentrations, has been proven beneficial to survival in liver malignancies^4,5^. Wang et al.^6^ have demonstrated the efficacy of HAIC using oxaliplatin and 5-fluorouracil, with a median progression-free survival (mPFS) and an mOS of 12.2 and 20.5 months, respectively, in a prospective phase II trial. However, the survival benefit of HAIC for pCCA patients is variable. Hence, there is a clinical need for reliable survival biomarkers that can be used to predict the effectiveness of HAIC in pCCA patients.

In cancer patients, lymphocytes are the most responsible immune cells and can eradicate tumor cells by inhibiting cell proliferation or migration^7^. However, tumor-associated neutrophils can modulate the extracellular matrix and suppress the cytolytic activity of immune cells^8^. The neutrophil-to-lymphocyte ratio (NLR) in peripheral blood tests has been used as a convenient inflammatory biomarker because of its association with system inflammation and tumor microenvironment. Numerous reports and meta-analyses have found that an elevated NLR before treatment is related to worse OS in several malignancies^9–11^. However, whether NLR is closely related to the survival of pCCA patients who received HAIC remains unclear.

Therefore, the purpose of the study was first to evaluate the neutrophil-to-lymphocyte ratio at baseline (bNLR) as a survival biomarker and then develop an applicable score based on serum inflammatory-tumor biomarkers to predict treatment outcomes for pCCA patients receiving first-line HAIC.

## Materials and methods

### Study design

This retrospective study was approved by the institutional review board of Peking University Cancer Hospital (approval protocol number: 2021KT144), and the requirement for informed consent was waived by the institutional review board of Peking University Cancer Hospital. All methods were performed in accordance with the Declaration of Helsinki. The data on pCCA patients from a single center for 10 consecutive years (January 2011 to January 2020) were reviewed.

The inclusion criteria were: (1) unresectable pCCA patients, including those with Blumgart T3 Stage lesions^12^, N2 lymph node metastasis, intrahepatic or distant metastasis, liver cirrhosis, or decreased liver function; (2) patients with a total bilirubin level less than 100 μmol/L and albumin level greater than 30 g/L prior to HAIC treatment; (3) patients who received first-line HAIC for at least two cycles using the 3cir-OFF regimen; (4) patients who underwent peripheral blood tests within 7 days before the first cycle of HAIC procedure; and (5) patients who had abdominal contrast-enhanced computed tomography (CT) or magnetic resonance (MR) imaging within 1 month before the first cycle of HAIC and after every two cycles.

The exclusion criteria were: (1) coexistent malignancies; (2) received systemic chemotherapy, radiation therapy, resection, and other previous local treatments; (3) received HAIC other than the 3cir-OFF regimen, concomitant to other local treatment or systemic treatment; (4) absence of tumor response evaluation; and (5) a follow-up period of less than 6 months.

**Figure 1:**
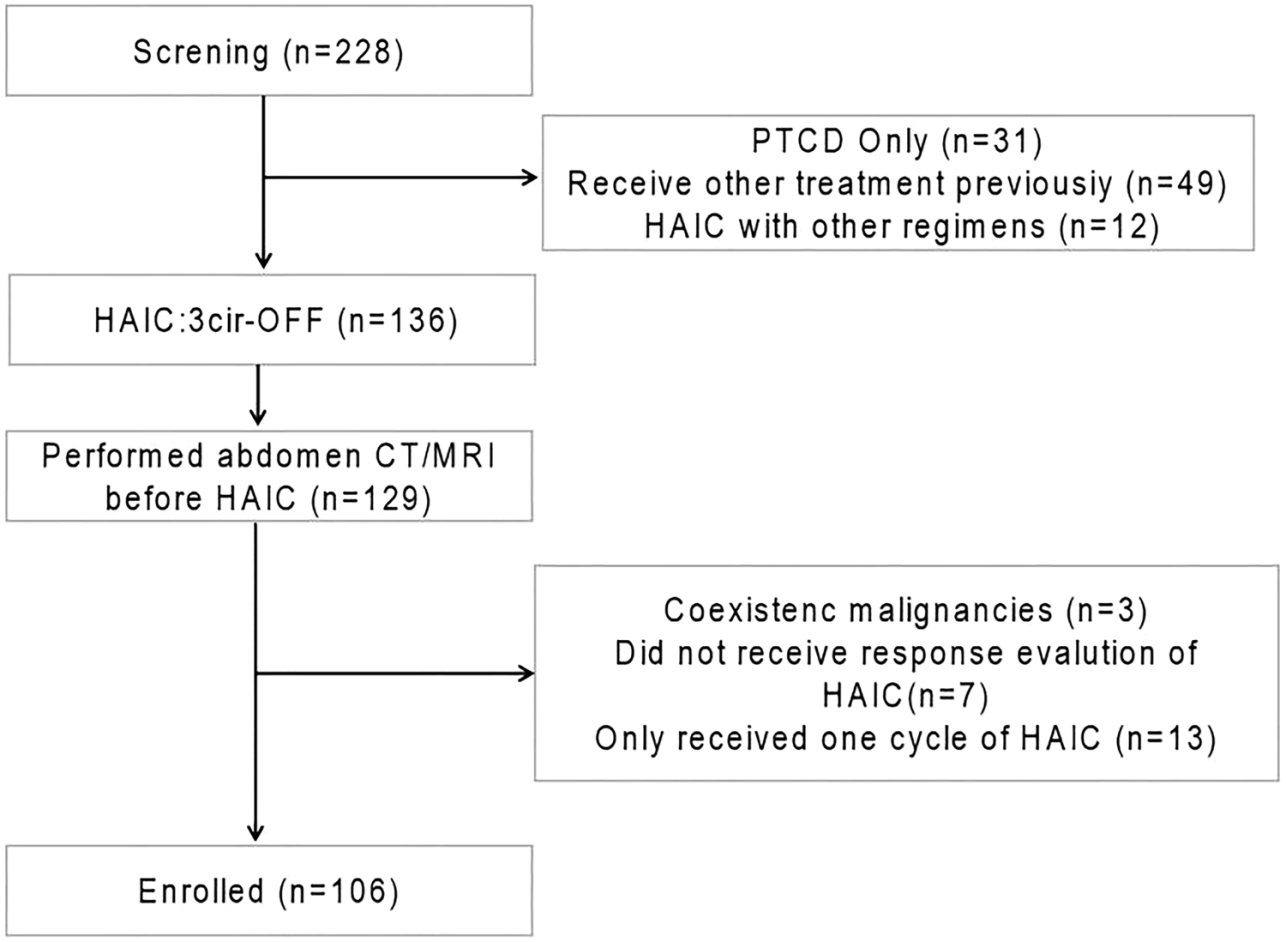
Patient flowchart. *HAIC* hepatic arterial infusion chemotherapy, *PTCD* percutaneous transhepatic cholangial drainage.

A total of 228 advanced pCCA patients were treated from January 2011 to January 2020, of which 31 patients only received percutaneous transhepatic cholangial drainage (PTCD), 49 patients had previously received systemic chemotherapy, radiation therapy, resection, or other local treatments, and 12 patients received HAIC with different regimens. Among the other 136 patients who received HAIC (3cir-OFF), 129 patients had abdominal imaging before the initiation of HAIC. Of the 129 patients, three had coexisting malignancies, seven did not receive a response evaluation of HAIC, and 13 patients only received one cycle of HAIC. Finally, 106 pCCA patients were enrolled (Fig. 1). According to the same inclusion and exclusion criteria, an additional 33 advanced pCCA patients who received first-line HAIC treatment at our center from January 2020 to January 2022 served as the validation cohort.

### HAIC procedure

HAIC was performed by percutaneous implantation of indwelling port catheter systems as previously described^13^. First, all extrahepatic arteries branching out from the hepatic artery, such as the right gastric artery and accessory left gastric artery, were embolized with 0.018-inch micro-coils. Second, hepatic arterial blood flow was redistributed using 0.018-inch micro-coils to convert multiple arteries into a single arterial blood supply in cases of variation involving multiple hepatic arteries. Finally, the catheter tip was fixed in the gastroduodenal artery, and a side hole was opened in the distal part of the common hepatic artery. Subsequently, the indwelling catheter was connected to the port. The HAIC regimen was 3cir-OFF, consisting of oxaliplatin (40 mg/m^2^ for 2 h), 5-fluorouracil (800 mg/m^2^ for 22 h), and folinic acid (200 mg/m^2^, 2 h from the beginning of 5-fluorouracil infusion) on days 1–3 every 4 weeks^14–19^. A maximum of six consecutive HAIC cycles were performed on patients not showing disease progression. Maintenance therapy with oral capecitabine was followed until tumor progression.

### Follow-up and assessments

Overall survival was calculated from the initiation of HAIC to death or last follow-up. PFS was defined as the period from the date of HAIC to disease progression, death, or last follow-up, whichever happened first. All patients underwent contrast-enhanced CT or MR imaging and routine laboratory studies at baseline. During the treatment phase, patients underwent regular laboratory studies, serum carcinoembryonic antigen (CEA) and carbohydrate antigen 19-9 (CA19-9) level evaluation, and CT or MR imaging after every two HAIC cycles and every 3 months during capecitabine treatment. Survival follow-up was conducted every 2 months by telephone call until September 1, 2021 (January 1, 2024, for the validation cohort), or until the occurrence of death/loss to follow-up.

### Statistical analysis

CEA and CA19-9 cutoff values were used similar to previous publications (CEA: 10 ng/mL; CA199: 200 U/mL)^5,6^. The cutoff values of other baseline variables were defined by the receiver operating characteristic (ROC) method. Univariate and multivariate analyses were performed by the Cox proportional hazards regression method. Variables with a *P*-value < 0.05 on univariable analysis were considered for multivariable analysis. Variables identified as independent predictors for OS were used to construct the survival scoring system. OS/PFS was assessed by Kaplan–Meier analysis. Differences with a *P*-value < 0.05 were considered statistically significant. All analyses were performed by SPSS v.23.0 software (IBM Corp, Armonk, NY, USA).

### Ethics declarations

This retrospective study was approved by the institutional review board of Peking University Cancer Hospital (approval protocol number: 2021KT144).

### Consent to participate

This retrospective study was approved by the institutional review board, and the requirement for informed consent was waived.

## Results

### Patients

All 106 pCCA patients received HAIC (3cir-OFF) as first-line treatment. The final follow-up was completed on September 1, 2021. The median follow-up time was 59.3 months. Until last follow-up, 82 (77.4%) patients died, and 80 (75.5%) patients had progressed. The patients received a median of five cycles (range, two to six cycles) of HAIC. Of the 106 patients (mean age, 60.0 ± 10.4 years), 69 (65.1%) were male and 37 (34.9%) were female. The number of patients with Child–Pugh class A and B was 37 (34.9%) and 69 (65.1%), respectively. Forty-four (41.5%) patients had hepatitis B virus (HBV). The number of patients with albumin-bilirubin (ALBI) grades 1, 2, and 3 was 24 (22.6%), 77 (72.6%), and 5 (4.7%), respectively. There were 56 (52.8%) patients in locally advanced stage, 19 (17.9%) had N1 lymph node metastasis, and 31 (29.2%) had N2 lymph node metastasis or distant metastasis. The cutoff value of bNLR was defined as 3.19 by the receiver operating characteristic (ROC) method (area under the curve, 0.638; sensitivity, 0.688; specificity, 0.655.) according to 1-year survival after HAIC. Patients in the whole cohort were divided into a low bNLR group (bNLR ≤ 3.19; n = 53) and high bNLR group (bNLR > 3.19; n = 53) for further analysis (Table 1). The characteristics of patients in the validation cohort are presented in Table S1.

**Table 1:** Summary of patient baseline characteristics.

Characteristic	Value	Characteristic	Value
Age (y)	60.0 ± 10.4	Macroscopic growth patterns	
Sex		PI (periductal infiltrating)	64 (60.4%)
Male	69 (65.1%)	MF (mass-forming)	42 (39.6%)
Female	37 (34.9%)	CEA (carcinoembryonic antigen) (ng/mL)	
HBV (hepatitis B virus) infection		< 10 ng/mL	80 (75.5%)
No	62 (58.5%)	> 10 ng/mL	22 (20.8%)
Yes	44 (41.5%)	Unknown	4 (3.8%)
Child–Pugh class		CA19-9 (carbohydrate antigen 19-9) (U/mL)	
A	37 (34.9%)	< 200 U/mL	35 (33.0%)
B	69 (65.1%)	> 200 U/mL	71 (67.0%)
ALBI (albumin-bilirubin) grade			
1	24 (22.6%)	ECOG (Eastern Cooperative Oncology Group) performance status	
2	77 (72.6%)	0	44 (41.5%)
3	5 (4.7%)	1	42 (39.6%)
Total bilirubin (μmol/L)		2	20 (18.9%)
Median	66.3	NLR (neutrophil-to-lymphocyte ratio)	
Range	7.6–100	Median	3.19
Albumin (g/L)		Range	0.78–24.55
Median	40.00	HAIC (hepatic arterial infusion chemotherapy) cycles	
Range	25.80–84.80	Median	5
Extent of disease		Range	2–6
Locally advanced	56 (52.8%)	Receipt of bevacizumab	
N1 lymph node metastasis	19 (17.9%)	No	86 (81.8%)
N2 or extrahepatic distant metastasis	31 (29.2%)	Yes	20 (18.9%)

### Relationship between bNLR, disease extent, and survival

**Figure 2:**
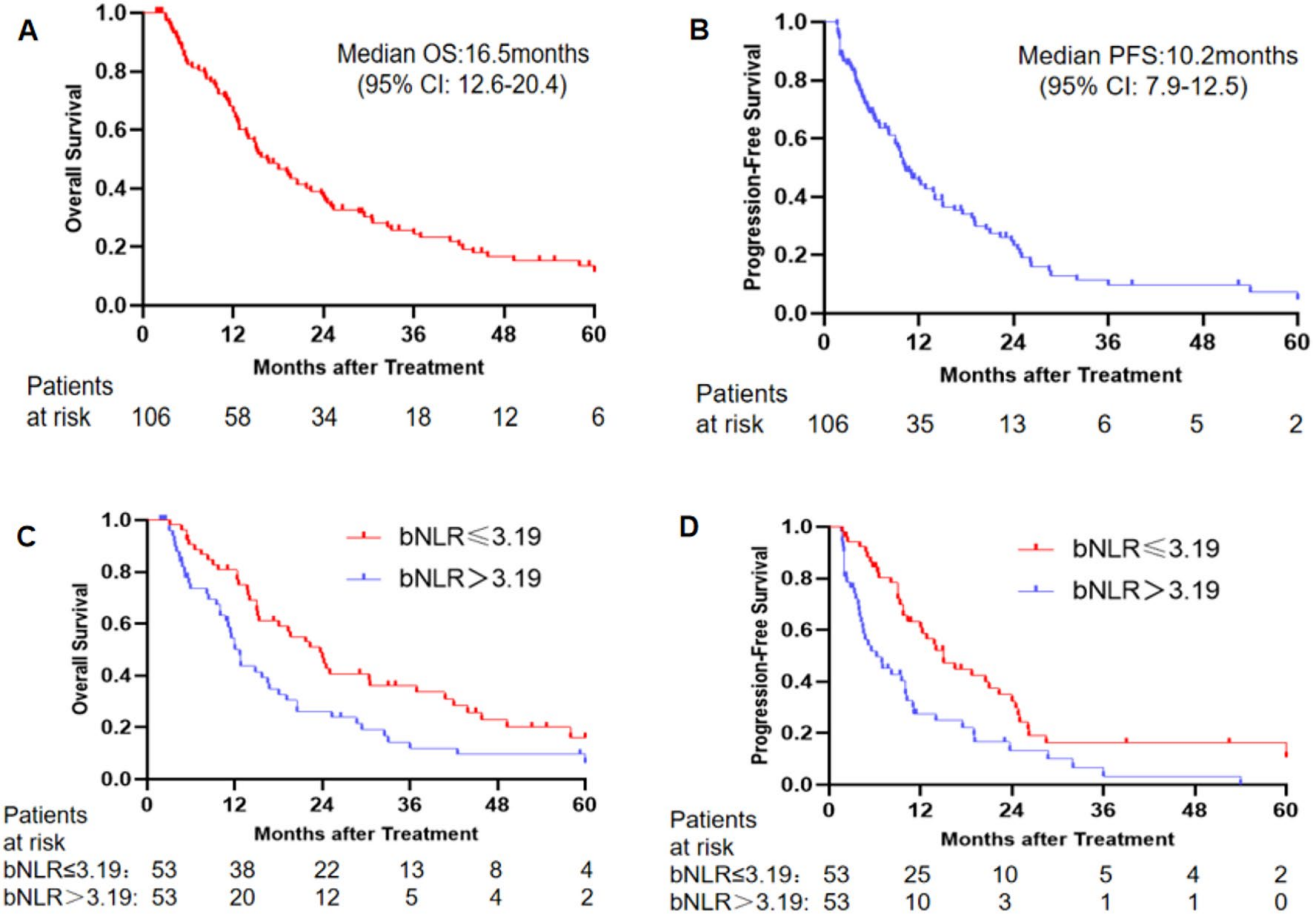
(**A**,**B**) Cumulative survival curves of hepatic arterial infusion chemotherapy for perihilar cholangiocarcinoma. (**C**,**D**) Cumulative survival curves of hepatic arterial infusion chemotherapy for perihilar cholangiocarcinoma low neutrophil-to-lymphocyte ratio at baseline and high neutrophil-to-lymphocyte ratio at baseline groups. *bNLR* neutrophil-to-lymphocyte ratio at baseline, *CI* confidence interval, *PFS* progression-free survival.

**Figure 3:**
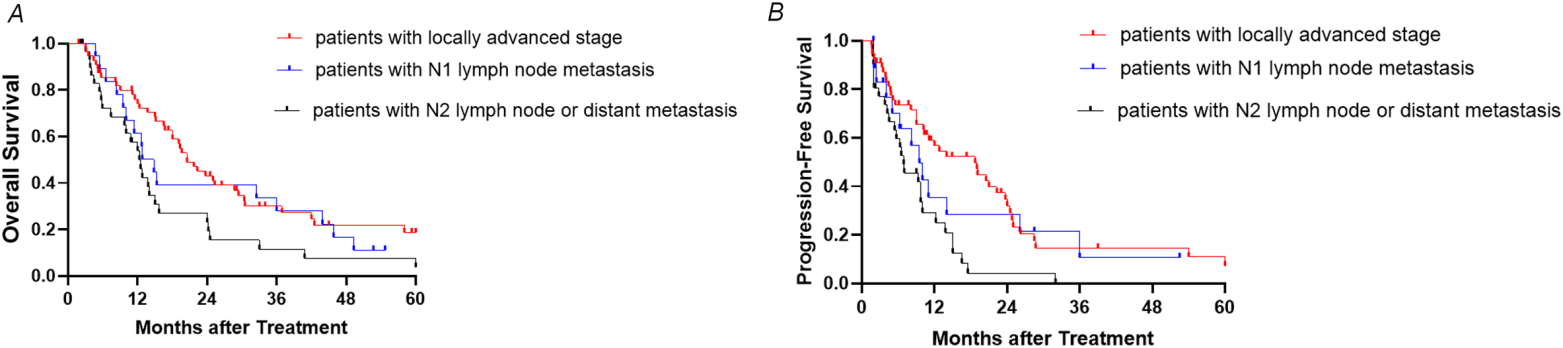
(**A**,**B**) Cumulative survival curves of hepatic arterial infusion chemotherapy for perihilar cholangiocarcinoma stratified by the extent of disease at different time points.

For these 106 pCCA patients, the median OS was 16.5 months [95% confidence interval (CI) 12.6–20.4] (Fig. 2A), and the median PFS was 10.2 months [95% CI 7.9–12.5] (Fig. 2B). The OS of patients in the low bNLR group (53 patients) was longer than that of the high bNLR group (53 patients) [23.7 months (95% CI 17.5–29.9 months) vs. 12.7 months (95% CI 11.2–14.2 months), hazard ratio (HR) 1.801, *P* = 0.007] (Fig. 2C). The PFS of the low bNLR group was also longer than that of the high bNLR group [15.0 months (95% CI 10.4–19.6 months) vs. 6.2 months (95% CI 2.5–9.9 months), HR 2.099, *P* = 0.001] (Fig. 2D). In addition, the mOS of patients with a locally advanced stage, N1 lymph node metastasis, and N2 lymph node metastasis or distant metastasis was 20.5 months (95% CI 16.0–25.0 months), 14.8 months (95% CI 10.5–19.1 months), and 12.3 months (95% CI 9.9–14.7 months), respectively (*P* = 0.029; Fig. 3A). The mPFS of patients with a locally advanced stage, N1 lymph node metastasis, and N2 lymph node metastasis or distant metastasis was 18.7 months (95% CI 10.3–27.1 months), 9.5 months (95% CI 6.3–12.7 months), and 6.9 months (95% CI 7.9–12.5 months), respectively (*P* < 0.001; Fig. 3B).

### Development of the NLCECA score (NLR, CEA, and CA19-9)

The extent of disease (HR 1.387, 95% CI 1.080–1.782, *P* = 0.010), macroscopic growth patterns (HR 1.895, 95% CI 1.219–2.948, *P* = 0.005), CEA > 10 ng/mL (HR 2.285, 95% CI 1.376–3.796, *P* = 0.001), CA19-9 > 200 U/mL (HR 2.457, 95% CI 1.475–4.093, *P* = 0.001), and NLR > 3.19 (HR 1.801, 95% CI 1.164–2.786, *P* = 0.007) were identified as risk factors for worse OS via univariate analysis. In the multivariate analysis, CEA > 10 ng/mL (HR 1.961, 95% CI 1.135–3.386, *P* = 0.016), CA19-9 > 200 U/mL (HR 2.264, 95% CI 1.320–3.883, *P* = 0.003), and NLR > 3.19 (HR 1.729, 95% CI 1.047–2.854, *P* = 0.032) were identified as independent predictors of worse OS (Table 2).

**Table 2:** Univariate and multivariate analyses of factors related to overall survival.

Characteristic	Univariate analysis		Multivariate analysis	
	HR (hazard ratio) (95% CI)	*P* value	HR (95% CI)	*P* value
Age	1.015 (0.991–1.040)	0.229		
Sex: male/female	0.901 (0.572–1.419)	0.651		
HBV infection: no/yes	0.964 (0.622–1.495)	0.870		
Child–Pugh class: A/B	0.807 (0.514–1.265)	0.349		
ALBI: 1/2/3	1.287 (0.816–2.030)	0.278		
Total bilirubin	1.004 (0.997–1.011)	0.264		
Albumin	0.981 (0.951–1.011)	0.203		
Extent of disease				
Locally advanced/N1/N2 or extrahepatic distant metastasis	1.387 (1.080–1.782)	0.010	1.247 (0.938–1.658)	0.128
Macroscopic growth patterns				
PI/MF	1.895 (1.219–2.948)	0.005	1.328 (0.773–2.281)	0.305
CEA level: < 10 ng/mL/> 10 ng/mL	2.285 (1.376–3.796)	0.001	1.961 (1.135–3.386)	0.016
CA19-9 level: < 200 U/mL/> 200 U/mL	2.457 (1.475–4.093)	0.001	2.264 (1.320–3.883)	0.003
ECOG performance status: 0/1/2	1.040 (0.775–1.394)	0.795		
Receipt of bevacizumab: no/yes	0.765 (0.422–1.385)	0.376		
bNLR level: low/high	1.801 (1.164–2.786)	0.007	1.729 (1.047–2.854)	0.032

Given that CEA, CA19-9, and bNLR were independent predictors for OS, a simple scoring method was developed to predict the survival of pCCA patients. Because the three factors had approximately similar hazard ratios (1.729, 1.961, and 2.264) in multivariable analysis of OS, one point was assigned to patients with NLR > 3.19, CEA > 10 ng/mL, and CA-199 > 200 U/mL. Thus, a patient could achieve either 0 (N = 18), 1 (N = 35), 2 (N = 41), or 3 points (N = 8). This simple score was named the NLCECA score (Table 3).

**Table 3:** The NLCECA score (NLR, CEA, and CA19-9).

Scoring systems	Score
NLR < 3.19, CEA < 10 ng/mL, and CA-199 < 200 U/mL	0
One of the three variables (NLR, CEA, and CA19-9) exceeded the cut off value	1
Two of the three variables (NLR, CEA, and CA19-9) exceeded the cut off value	2
NLR > 3.19, CEA > 10 ng/mL, and CA-199 > 200 U/mL	3

### Relationship between the NLCECA score and survival

**Figure 4:**
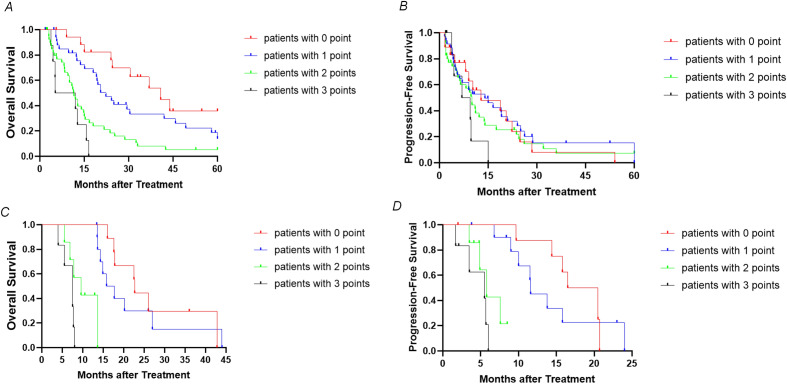
(**A**,**B**) Cumulative survival curves of hepatic arterial infusion chemotherapy for 106 perihilar cholangiocarcinoma patients at different time points. (**C**,**D**) Cumulative survival curves of hepatic arterial infusion chemotherapy for perihilar cholangiocarcinoma in the validation cohort at different time points.

The mOS of patients with 0, 1, 2, 3 points were 40.8 months (95% CI 25.3–56.3 months), 22.3 months (95% CI 16.0–28.6 months), 11.5 months (95% CI 9.5–13.5 months), and 5.2 months (95% CI 0–14.8 months), respectively (*P* < 0.001; Fig. 4A). The mPFS of patients with 0, 1, 2, 3 points were 20.5 months (95% CI 6.5–34.5 months), 14.0 months (95% CI 9.3–18.7 months), 6.5 months (95% CI 1.2–11.8 months, and 5.1 months (95% CI 3.4–6.8 months), respectively (*P* < 0.001; Fig. 4B). When including the NLCECA score in the multivariate analysis, the NLCECA score was the sole independent predictor of survival, and the risk of survival decreased by 111.9% for each 1-point increase in the NLCECA score (Table 4).

**Table 4:** Multivariate analyses of factors related to overall survival.

Variable	Multivariate analysis	
	Hazard ratio (95% confidence interval)	P-value
NLCECA score for (each one-point increase)	2.119 (1.595–2.816)	< 0.001

*Extent of disease, macroscopic growth patterns, carcinoembryonic antigen (CEA) level, carbohydrate antigen 19-9 (CA19-9) level, neutrophil-to-lymphocyte ratio (NLR) level, and NLCECA score were included in the initial multivariate analysis; extent of disease (*P* = 0.055), macroscopic growth patterns (*P* = 0.126), CEA level (*P* = 0.783), CA19-9 level (*P* = 0.894), and NLR level (*P* = 0.820) lost their significance and were therefore removed.

The usefulness of the NLCECA score was further validated in another 33 advanced pCCA patients who received first-line HAIC. The thirty-three patients in the validation cohort consisted of nine patients with 0 points, 11 patients with 1 point, seven patients with 2 points, and six patients with 3 points. For these 33 pCCA patients in the validation cohort, the median OS was 15.8 months (95% CI 12.7–18.9 months), and the median PFS was 11.5 months (95% CI 8.5–14.5 months). A higher NLCECA score in the validation cohort also indicated dismal survival. The mOS of patients with 0, 1, 2, and 3 points was 22.7 months (95% CI 22.1–23.3 months), 15.8 months (95% CI 11.5–20.1 months), 9.6 months (95% CI 5.0–14.2 months), and 7.5 months (95% CI 5.2–9.8 months), respectively (*P* < 0.001; Fig. 4C). The mPFS of patients with 0, 1, 2, and 3 points was 16.5 months (95% CI 12.2–20.8 months), 11.6 months (95% CI 11.3–11.9 months), 5.8 months (95% CI 4.0–7.6 months), and 5.5 months (95% CI 1.3–9.7 months), respectively (*P* < 0.001; Fig. 4D).

### Treatment toxicity

As shown in Table S2, the most common adverse event was nausea, which occurred in 65 (61.3%) patients. No treatment-related deaths occurred during first-line HAIC treatment. Additionally, 31 patients (29.2%) incurred Grade 3 or 4 adverse events. Elevated ALT/AST were the most common Grade 3 or 4 adverse events, occurring in 16 (15.1%) patients.

## Discussion

This retrospective study demonstrated NLR as a biomarker of survival for advanced pCCA patients receiving HAIC. Based on NLR, CEA, and CA19-9, which were identified as independent predictors of survival of pCCA after HAIC treatment, a simple and easily applicable clinical serum inflammatory-tumor biomarker system, the NLCECA score, consisting of NLR, CEA, and CA19-9, was established. When it was incorporated, the NLCECA score was the sole independent predictor of survival, and the risk of survival decreased by 111.9% for each 1-point increase in NLCECA score.

It is usually difficult to perform a core needle biopsy to obtain pCCA tissues due to its particular perihilar anatomical site and its periductal infiltration growth pattern along the bile duct wall. Most pathological diagnoses of advanced pCCA are based on cytopathology. Hence, for patients with advanced unresectable pCCA, there is a need for biomarkers of survival features. CEA and CA19-9 are widely used traditional prognostic biomarkers for pCCA. However, for the clinical application of CEA or CA19-9 alone, only 30% of CCA patients exhibit elevated CEA levels^20^ and CA19-9 is often falsely elevated in benign biliary disease or cholangitis^21^. Thus, a single indicator of CEA or CA19-9 may not well reflect the prognosis of pCCA patients.

Inflammation, which is one of the seven essential characteristics of tumors, can contribute to cancer progression^22^. CCA is also associated with biliary tract inflammation. Most cases of CCA arise during chronic infection of the biliary tree such as cholelithiasis, infection with liver fluke *Clonorchis sinensis*, or primary sclerosing cholangitis^23^. Various inflammatory biomarkers such as NLR, lymphocyte-to-monocyte ratio, and platelet-to-lymphocyte ratio could effectively reflect the degree of inflammation and immune response and are recommended as predictive biomarkers for cancer patients^24,25^. Of the various inflammatory biomarkers, NLR has drawn a large amount of attention because of the function and characteristics of neutrophils and lymphocytes in tumor development. Previous studies have revealed that high neutrophil counts are associated with immunosuppression conditions^26^. Lymphocytes recognize and eliminate tumor cells, as well as inhibit cancer cell proliferation by producing cytokines^27^. Therefore, low lymphocyte counts may be related to impaired adaptive immunity activation^28^. Thus, high NLR levels serve as a potential biomarker of worse prognosis, which is similar to the results of our study.

The prognosis of advanced pCCA is associated with the proliferation and metabolism of the tumor, as well as the local and systemic inflammatory immune status^29^. CEA and CA19-9 are the main protein products of tumor metabolism secreted into the peripheral blood during tumor proliferation and progression^30,31^ and have been used as prognosis-related biomarkers in biliary tract cancer^32^. NLR in peripheral blood is associated with tumor immune microenvironment and system inflammation, which has been used as a convenient inflammatory biomarker^9–11^. In our study, a new serum inflammatory-tumor biomarker system, the NLCECA score based on NLR, CEA, and CA19-9, was established, and it accurately reflected the tumor growth and proliferation level, as well as the body and tumor inflammatory immune status. When it was incorporated, the NLCECA score was the sole independent predictor of survival, with the risk of survival decreasing by 111.9% for each 1-point increase in the NLCECA score. As a liquid marker, it could aid in the selection of advanced pCCA patients for first-line HAIC in clinical practice. Moreover, due to the easily accessible clinical laboratory test indexes adopted in this score, it is convenient for clinical application.

Our research has several limitations. First, because this was single-center retrospective research, results require further validation in more extensive, multi-center studies. Second, the association of neutrophils to each subgroup lymphocyte ratio with the survival of pCCA patients was not further explored in this study.

In summary, as an immune and inflammatory indicator, low bNLR is an independent predictor of better survival of pCCA after HAIC treatment. A new established serum inflammatory-tumor biomarker system, the NLCECA score, consisting of NLR, CEA, and CA199, may be a reliable survival prediction system for advanced pCCA after HAIC treatment. Prospective validation of the NLCECA score is also warranted.

### Supplementary Information

Supplementary Tables.

## Data Availability

The data that support the findings of this study are available from the corresponding author upon reasonable request.
