# Supplementary material for: NLCECA score: a serum inflammatory-tumor biomarker score to predict survival of advanced perihilar cholangiocarcinoma after hepatic arterial infusion chemotherapy
Source: Sci Rep. 2024 Feb 23;14:4466. doi: 10.1038/s41598-024-53883-7 (PMC10891088; doi:10.1038/s41598-024-53883-7)
Supplement: Supplementary file 1 — Supplementary Tables. [file 41598_2024_53883_MOESM1_ESM.docx]

**NLCECA score: A serum inflammatory-tumor biomarker score predict survival of advanced perihilar cholangiocarcinoma after hepatic arterial infusion chemotherapy**

Shjie Fu^1^, Jie Li^2^, Hua Fan^3^, Kanglian Zheng^1^, Boyu Leng^4^, Guang Cao^1^, Liang Xu^1^, Yujie Zhong^1^, Chuanxin Niu^1^ & Xiaodong Wang^1🖂^

^1^Key Laboratory of Carcinogenesis and Translational Research (Ministry of Education/Beijing), Department of Interventional Oncology, Peking University Cancer Hospital & Institute, Beijing 100142, China. ^2^Center for Medical Device Evaluation, National Medical Products Administration, Beijing 100053, China. ^3^Key Laboratory of Carcinogenesis and Translational Research (Ministry of Education/Beijing), Department of Cancer Epidemiology, Peking University Cancer Hospital & Institute, Beijing 100142, China. ^4^Hebei North University, Hebei 075000, China. ^🖂^email: xiaodongw75@yahoo.com

Shjie Fu and Jie Li contributed equally to this work.

| **Characteristic** | **Value** | **Characteristic** | **Value** |
| --- | --- | --- | --- |
| Age (y) | 62.0 ± 11.9 | Macroscopic growth patterns |  |
| Sex |  | PI (periductal infiltrating) | 20 (60.6%) |
| Male | 22 (66.7%) | MF (mass-forming) | 13 (39.4%) |
| Female | 11 (33.3%) | CEA (carcinoembryonic antigen) (ng/mL) |  |
| HBV (hepatitis B virus) infection |  | <10 ng/mL | 26 (78.8%) |
| No | 62 (58.5%) | >10 ng/mL | 7 (21.2%) |
| Yes | 44 (41.5%) |  |  |
| Child-Pugh class |  | CA19-9 (carbohydrate antigen 19-9) (U/mL) |  |
| A | 17 (51.5%) | <200 U/mL | 15 (45.5%) |
| B | 16 (48.5%) | >200 U/mL | 18 (54.5%) |
| ALBI (albumin-bilirubin) grade |  |  |  |
| 1 | 8 (24.2%) | ECOG (Eastern Cooperative Oncology Group) performance status |  |
| 2 | 23 (69.7%) | 0 | 22 (66.7%) |
| 3 | 2 (6.0%) | 1 | 9 (27.3%) |
| Total bilirubin (μmol/L) |  | 2 | 2 (6.1%) |
| Median | 49.2 | NLR (neutrophil-to-lymphocyte ratio) |  |
| Range | 11.7–100 | Median | 2.91 |
| Albumin (g/L) |  | Range | 0.52–50.59 |
| Median | 40.3 | HAIC (hepatic arterial infusion chemotherapy) cycles |  |
| Range | 30.2-49.2 | Median | 4 |
| Extent of disease |  | Range | 2–6 |
| Locally advanced | 12 (36.4%) | Receipt of bevacizumab |  |
| N1 lymph node metastasis | 12 (36.4%) | No | 23 (69.7%) |
| N2 or extrahepatic distant metastasis | 9 (27.3%) | Yes | 10 (30.3%) |

**Table S1.** Summary of validation cohort patients baseline characteristics.

| Event (%) | Any grade | Grade 3 or 4 |
| --- | --- | --- |
| Any adverse event | 95 (89.6%) | 31 (29.2%) |
| Gastrointestinal events |  |  |
| Nausea | 65 (61.3%) | 4 (3.8%) |
| Vomiting | 40 (37.7%) | 3 (2.8%) |
| Diarrhea | 32 (30.2%) | 6 (5.7%) |
| Stomach ache | 49 (46.2%) | 2 (1.9%) |
| Hepatotoxicity |  |  |
| Elevated ALT/AST | 44 (41.5%) | 16 (15.1%) |
| Hyperbilirubinemia | 37 (4.9%) | 7 (6.6%) |
| Hematotoxicity |  |  |
| Leukopenia | 46 (43.4%) | 9 (8.5%) |
| Thrombocytopenia | 42 (39.6%) | 11 (10.4%) |
| Neutropenia | 24 (22.6%) | 6 (5.7%) |
| Anemia | 38 (35.8%) | 8 (7.5%) |
| Fever | 27 (25.4%) | 2 (1.9%) |

**Table S2:** Toxic effects encountered during treatment.
